# SPPIPred: Stacking-based ensemble learning model for identification of protein-protein interaction

**DOI:** 10.1371/journal.pone.0353199

**Published:** 2026-07-17

**Authors:** Md. Ashikur Rahman, Md. Mamun Ali, Md. Shohidullah, Kawsar Ahmed, Francis M. Bui, Li Chen, Mohammad Ali Moni

**Affiliations:** 1 Department of Software Engineering (SWE), Daffodil International University (DIU), Daffodil Smart City (DSC), Birulia, Savar, Dhaka, Bangladesh; 2 Division of Biomedical Engineering, University of Saskatchewan, Saskatoon, Canada; 3 Health Informatics Research Lab, Department of Computer Science and Engineering, Daffodil International University, Dhaka, Bangladesh; 4 Department of Electrical and Computer Engineering, University of Saskatchewan, Saskatoon, Canada; 5 AI & Digital Health Technology, Artificial Intelligence & Cyber Future Institute, Charles Sturt University, Bathurst, New South Wales, Australia; 6 AI & Digital Health Technology, Rural Health Research Institute, Charles Sturt University, Orange, New South Wales, Australia; Albert Einstein College of Medicine, UNITED STATES OF AMERICA

## Abstract

Protein-protein interactions (PPIs) are essential for various biological functions and are crucial in drug discovery, signaling pathways, and network reconstruction. This study presents SPPIPred, an advanced machine learning-based model designed for precise PPI prediction. The SPPIPred model was constructed using five feature extraction methods: Pseudo amino acid composition (PAAC), Composition transition distribution (CTDC), Dipeptide composition (DPC), Word2Vec, and FastText. Among these, FastText emerged as the most effective for encoding protein sequences. Despite the application of feature selection techniques, the analysis revealed that the original raw feature dimensions yielded superior results compared to the selected features. The model used seven machine learning classifiers, including Decision Tree (DT), Extra Trees Classifier (ETC), CatBoost (CAT), XGBoost (XGB), LightGBM (LGBM), Random Forest (RF), and the stacking model named SPPIPred. SPPIPred demonstrated exceptional accuracy rates of 0.9989 in the *H pylori* dataset and 0.9991 in the *S cerevisiae* dataset, with Matthews correlation coefficients (MCC) of 0.9982 and 0.9979, respectively. These findings highlight the effectiveness and reliability of the SPPIPred model, offering valuable insights to researchers in the field of bioinformatics and improving applications within bioengineering and pharmaceutical development.

## 1. Introduction

Protein is an essential component of all cells in the human body’s organs. It is crucial for cell activity as well as for the structure, mechanism, and control of the body’s systems. Protein-protein interaction (PPI) plays a vital role in biological functions as well as metabolism, cell-to-cell signaling, and monitoring of functional changes in the cell. PPI is also involved in in other biological processes, including DNA replication, cellular metabolism, control of gene expression, cell signaling, biogenesis, and immunological response. In recent years, one of the hot topics in system biology is PPI. Researchers can benefit from studying PPI sites in a variety of fields, including the construction of interacting protein networks, the new design of drugs, and the gene regulation pathways [1,2]. Machine learning (ML) techniques are now widely used in biomedical, system biology, and bioinformatics research. ML has overcome the traditional laboratory-based experimental shortcomings. For predicting PPI, ML has been widely used [3]. It reduces the influence of errors in the lab-based experiment on PPI prediction, whereas improving the overall current computational model of protein interactions. Through the development of protein interaction networks, improved computational approaches can uncover the origins and pathophysiology of the disease, in addition to providing more clarity to canonical pathways [4].

Protein sequence feature extraction is a crucial stage for utilizing ML efficiently to predict PPI sites [5–7]. Researchers have conducted numerous studies in the past few years to encode and extract features from protein sequences. But there has been some scope for new work on extracting and encoding features. Most studies use a variety of feature extraction techniques, including network-based, position-based, structure-based, sequence-based, and evolution-based methodologies [4]. Zhang et al. (2019) used a variety of feature extraction techniques, such as protein sequence coding, 3D-1D scores, and conservation scores [8]. Murakami et al. (2010) performed predicted accessibility (PA) and position-specific scoring matrix (PSSM) feature encoding techniques [9]. Wang et al. (2021) perform different feature encoding techniques such as position-specific scoring matrix (PSSM), accessible surface area (ASA), pseudo-amino acid composition (PAAC), and hydropathy index [2]. Yu et al. (2021) applied physicochemical-based (PAAC, Moreau-Broto and Moran), sequence-based (MMI and CTD) and evolution-based (PSSM, AAC-PSSM and DPC-PSSM) feature extraction approaches [3]. Dhole et al. (2014) extract characteristics through PSSM, predict relative solvent accessibility (PRSA) and average cumulative hydropathy (ACH) [10]. PseAAC, the conjoint triad (CT), the autocorrelation descriptor (AD), and the extractor of local descriptor characteristics (LD) are integrated by Chen et al. (2019) [11].

The researchers carried out numerous studies for the development of PPI prediction models. Consequently, choosing the appropriate classifiers is essential for the prediction of PPI. Chen et al. (2020) have shown that ensemble classifiers have an accuracy of 94.64% on the S. cerevisiae dataset and an accuracy of 89.27% on the H. pylori dataset [4]. This performance can be improved with less computational complexity. Yu et al. (2021) proposed (Gc-Forest PPI) a deep forest model based on cascade architecture using XGBoost (XGB), Random Forest (RF) and Extremely Randomized Trees (ERT) and obtained a precision of 95.44% in the S. cerevisiae dataset. For H. pylori, the accuracy was 89.26% [3]. Wang et al. (2021) used XGB-based ML algorithms to build PPISP-XGBoost, and the training accuracy was 85.4% along with the highest independent test accuracy of 85.8%. Murakami et al. (2010) used Naive Bayes (NB) classifiers to build PPI prediction and achieved an accuracy of 83.3% [9]. Chen et al. (2019) proposed a PPI prediction model, LightGBM-PPI, that performed with an accuracy of 89.03% on the H. pylori data set and 95.07% on the S. cerevisiae data set [11]. Göktepe and Kodaz (2018) proposed an effective sequence-based combined method for PPI prediction with the highest accuracy of 93.45% [12]. Wei et al. (2015) predicted the PPI prediction model using cascade random forest (CRF) and RF, with results of 66.20% and 61.00% accuracy, respectively [13]. Wang et al. (2019) proposed a model based on the synthetic minority oversampling technique (SMOTE) with an accuracy of 77.1% and 77.7% in two different data sets for PPI prediction [14].

The primary objective of this study is to improve the accuracy of existing state-of-the-art protein-protein interaction (PPI) prediction models. To achieve this, the research introduces word embedding techniques to effectively encode the features of protein sequences, thereby improving the representation of these sequences for analysis.

In this research, a machine learning-based PPI prediction model is proposed, utilizing stacked ensemble classifiers. The model demonstrates exceptional performance across all evaluation metrics, confirming its effectiveness in predicting PPIs. This study contributes to the field by highlighting the benefits of applying advanced machine learning techniques and word embedding methods, which have gained traction among researchers in recent years. In general, the findings aim to advance current methodologies in PPI prediction and provide a more reliable framework for future bioinformatics research. The contributions of this study are as follows:

1. We systematically combined different best-fit heterogenous baseline classifiers for robust generalization across two different datasets.
2. We showed that the proposed model benefits more from word embedding features than do the other baseline classifiers.
3. Our proposed stacking method provides a higher F1-score and MCC value along with improved accuracy, which supports the robustness of the proposed model.

## 2. Materials & methods

**Fig 1:**
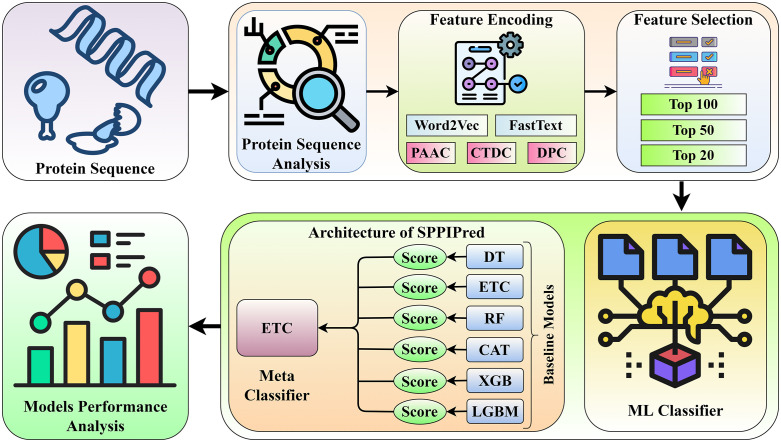
Working methodology of this research to build an SPPIPred PPI prediction model.

This section outlines the research methodology, detailing each stage from data collection to the development of the proposed SPPIPred model. The following subsections provide an overview of the dataset’s characteristics, feature encoding techniques, model construction, feature selection processes, and performance evaluation metrics. The detailed working procedure of this study is illustrated in Fig 1.

### 2.1. Data set description

To develop the PPI prediction model, two separate protein datasets were acquired. The first dataset was obtained from the DIP database and consists of 5,594 pairs of interacting proteins and 5,594 pairs of non-interacting proteins, specifically from *Saccharomyces cerevisiae* [15,16]. The second dataset, referenced from Martin, S., Roe, D., and Faulon, J.L. (2005), contains 1,458 interacting protein pairs and an equal number of non-interacting pairs, focusing on *Helicobacter pylori* [17]. The following subsection elaborates on the methodology implemented after collecting these datasets, detailing the procedures undertaken to construct and assess the PPI prediction model.

### 2.2. Features encoding techniques

ML algorithms cannot be trained directly on raw protein sequences, as these consist of character strings representing amino acid residues. For that reason, researchers must encode protein sequences and represent them as numeric values that are readable by the ML algorithms [18].

#### 2.2.1. Pseudo-amino acid composition (PAAC).

Chou (2001) first introduced PAAC for the extraction of amino acid sequences into numeric values [19]. It is still to be applied to enhance the domains of bioinformatics [20–25]. Incorporating sequence-based information into its pseudo-modules, PAAC analyses and expresses the frequency of each amino acid composition [26,27].


$$\begin{array}{c} x_{\mu }=\left\{ \begin{array}{cc} \frac{f_{\mu }}{\sum_{\mu =\text{1}}^{\text{2}\text{0}}f_{\mu }+\omega \sum_{k=\text{1}}^{\lambda }\tau_{k}},\; & \text{1}\leq \mu \leq \text{2}\text{0}\\ \frac{\omega \tau_{\mu -\text{2}\text{0}}}{\sum_{\mu =\text{1}}^{\text{2}\text{0}}f_{\mu }+\omega \sum_{k=\text{1}}^{\lambda }\tau_{k}},\; & \text{2}\text{1}\leq \mu \leq \text{2}\text{0}+\lambda \end{array}\right. \end{array}$$ (1)

A weighting factor of ω=0.05 is used in this study. The PAAC combines both traditional amino acid composition data and sequence-order information to extract features from protein sequences by recognizing each protein sequence’s large or global composition of all amino acids, and how they relate locally with other amino acids within a sequence [28].

#### 2.2.2. CTDC.

The CTDC feature extractor uses the structure property of the composition transition distribution (CTD) feature extractor to calculate the incidence of an estimated ratio of a specific amino acid property for the protein sequence [29]. The CTD characteristic extractor within a protein sequence indicates the structure of amino acid concentrations of structural or physicochemical characteristics [30]. Three components can be produced by the analysis of composition descriptors.


$$\begin{array}{c} \begin{array}{c} Composition(r)=\frac{L(r)}{L},\;r\in \left\{P,\;N,H\right\} \end{array} \end{array}$$ (2)

The frequency of *r* is expressed as L(r) in the above equation. The length of the protein sequence is 29 in the formula, which is indicated by seven ‘P’, ten N, twelve H. As a result, the values for “P,” “N,” and “H “ are 7/29 = 0.2414, 10/29 = 0.3448, and 12/29 = 0.4138 [31–33].

Based on the paired situation mentioned by the transition descriptor, the protein sequence transforms into a substituted sequence. On the substituted sequence, the transition descriptor is the dipeptide frequency values.


$$\begin{array}{c} \begin{array}{c} \text{Transition}(r,s)=\frac{L(r,s)+L(s,r)}{L-\text{1}},\;r,s\in \left\{(P,N),\;(N,H),\;(H,\;P)\right\} \end{array} \end{array}$$ (3)

The frequency of rs is represented as L(r,s) in this formula. The percentage frequencies of ‘P’ to ‘H’ or ‘H’ to ‘P’, ‘H’ to ‘N’ or ‘N’ to ‘H’, ‘N’ to ‘P’ or ‘P’ to ‘N’ are respectively 0.25, 0.1768, and 0.1071 [33–35].

The CTDC feature extractor can be calculated using the following formula.


$$\begin{array}{c} \begin{array}{c} CTDC=\frac{\text{NRi}}{L},\;Ri\in \left\{\text{Positive},\text{ Negative},\text{ Neutral}\right\} \end{array} \end{array}$$ (4)

For the above equation, neutral = {A, N, C, Q, G, H, I, L, M, F, P, S, T, W, Y, V}, positive = {K, R}, and negative = {D, E}, By using the CTDC feature extractor, the dimension of the feature vector is 39 [29,34].

#### 2.2.3. Dipeptide composition.

The protein sequence is transformed into a 2D array by the composition of the dipeptides (DPC), which contains the frequency of appearance of every pair of amino acids within the sequence (20 × 20) [35]. The following formula is followed by the DPC extractor feature for calculation.


$$\begin{array}{c} \begin{array}{c} A(t)=\frac{N(t)}{C} \end{array} \end{array}$$ (5)

In the above equation, t = 1,2,3, 4…0.400, the dipeptide is denoted by N. C, and t denote the total number of probable dipeptides [36].

#### 2.2.4. Word2Vec.

One of the most widely used word embedding techniques is word2vec. It is designed to turn words into randomized representations of numerical vectors. Word2vec encodes words into vectors that depict word associations and their interpretation. In 2013, Google introduced Word2Vec as a word embedding tool using deep learning (DL) [37]. Word2Vec uses a one-layer neural network containing parameters such as context windows, vector dimensions, etc. to produce vectors. There are mainly two types of embedding: continuous-bag-of-words (CBOW) and skip-gramme. Once a specific word is provided, CBOW estimates the embedding of the word; on the contrary, based on the size of the specified context window, Skip-gramme estimates the embedding of the word [38].

#### 2.2.5. FastText.

In 2016, a group of AI researchers from Facebook proposed a word embedding method called FastText [39]. FastText divides words into many n-grammes rather than giving the neural network single words. A character n-gramme is a sequence of n pieces from a specific sample of a letter or word. Bigram, trigram, etc. could be the case. The CBOW and skip-gramme techniques are also supported by the FastText word embedding. FastText is more advanced than Word2Vec for word embedding [40].

### 2.3. Proposed SPPIPred Development

The SPPIPred model is a stacking-based ensemble framework designed to efficiently predict protein-protein interactions (PPIs) using a range of robust machine learning classifiers. Several base classifiers are used in the SPPIPred architecture to capture various predicted patterns. The outputs are then combined and refined by a meta classifier. The SPPIPred operating method is broken down here. The SPPIPred model employs six baseline classifiers for the initial learning layer. Decision Tree (DT) is a simple tree-based model that captures nonlinear interactions in data [41]. Extra Tree Classifier (ETC) is a variant of decision trees with randomised splits that enhances variance reduction [42,43]. Categorical Boosting, or CatBoost (CAT), is a gradient boosting model that handles categorical data efficiently [44,45]. XGBoost is an optimized gradient boosting model focused on speed and performance [46,47]. LightGBM (LGBM) is a highly efficient gradient-boosting framework that uses a leaf-wise growth strategy for better accuracy [48]. Random Forest (RF) is an ensemble of decision trees that improves predictive stability and accuracy [49]. Each of these classifiers is trained independently on the input feature set to predict the probability of protein-protein interaction.

***Train Baseline Model:*** Given a dataset (*X*, *Y*), where *X* represents the characteristic vectors and *Y* represents the target indicating protein interaction, each base classifier Ci is trained on (*X*, *Y*) to predict probabilities of the interaction $P_{C_{i}}(X)$.

The prediction of each classifier *C*_*i*_ for a data sample *X*_*j*_ can be expressed as


$$\begin{array}{c} \begin{array}{c} P_{C_{i}}\left(X_{j}\right)=\;C_{i}\left(X_{j}\right) \end{array} \end{array}$$ (6)

Where, $P_{C_{i}}\left(X_{j}\right)$ is the probability of interaction predicted by the 𝑖-th base classifier for sample $X_{j}$.

***Stacking and Training the Meta Model:*** After training the base classifiers, the predictions for each sample are transformed into a new set of features *Z*, where each feature is a prediction from a baseline classifier. For an input sample *X*_*j*_, the transformed feature vector *Z*_*j*_ is:


$$\begin{array}{c} \begin{array}{c} Z_{j}=\left[P_{C_{\text{1}}}\left(X_{j}\right),\;P_{C_{\text{2}}}\left(X_{j}\right),\;\ldots ,\;P_{C_{\text{6}}}\left(X_{j}\right)\right] \end{array} \end{array}$$ (7)

This stacking process results in a transformed dataset (*Z*, *Y*) where each *Z*_*j*_ represents the combined predictive output of the baseline classifiers.

The ETC is used as the meta-classifier to learn from the combined output *Z* generated by the base classifiers. The meta-classifier is trained on *(Z, Y)* to produce the final prediction for the protein-protein interaction probability. The prediction of the meta-classifier for a sample *X*_*j*_ is given by:


$$\begin{array}{c} \begin{array}{c} P_{meta}\left(X_{j}\right)=ETC\left(Z_{j}\right)=ETC\left(P_{C_{\text{1}}}\left(X_{j}\right),\;P_{C_{\text{2}}}\left(X_{j}\right),\;\ldots ,\;P_{C_{\text{6}}}\left(X_{j}\right)\right) \end{array} \end{array}$$ (8)

Where, $P_{meta}\left(X_{j}\right)\;$represents the final probability of interaction as predicted by SPPIPred.

***SPPIPred Prediction:*** The decision to classify *X*_*j*_ as interacting or non-interacting is based on a threshold applied to the final probability $P_{meta}\left(X_{j}\right)$:


$$\begin{array}{c} \begin{array}{c} {\hat{Y}}_{j}=\;\left\{ \begin{array}{c} @c\text{1}\;if\;P_{meta}\left(X_{j}\right)\geq Threshold\\ \text{0}\;Otherwise \end{array}\right.\; \end{array} \end{array}$$ (9)

Here, $X_{j}$ denotes the predicted label for the sample, with the threshold typically optimized during model validation to balance sensitivity and specificity. Using stacking, SPPIPred combines the strengths of several classifiers, enhancing the PPI prediction accuracy by enabling the meta classifier to learn intricate relationships from the initial predictions. This architecture demonstrates the subtleties of each classifier, as well as the combined predictive power, resulting in strong performance for tasks involving protein-protein interactions [50–53].

### 2.4. Evaluation of model performance

One of the essential steps of any ML approach is to evaluate the performance of the classification algorithms. Although many performance metrics exist, this study employed six metrics to evaluate the ML models. After applying the performance evaluation, we have found the best model for our research. For evaluating the performance of the models in this study, we use k-fold cross validation (CV). k-fold CV is a method used to assess how well a model generalizes by dividing the dataset into k-folds. For every iteration of the algorithm, one-fold is used for testing and the remaining folds are used for training. Once all iterations have been completed, the average predicted accuracy is reported across all folds, providing a reliable estimate of model performance [54,55]. In this study, 10-fold CV is used for assessing the performance of the models.

Accuracy is the ratio of correctly classified instances to the total number of instances. When the desired feature classes in the data are relatively equal, accuracy is an acceptable statistic [56]. If we want to be certain in our prediction model, precision is an acceptable evaluation metric outcome. In terms of precision, all predicted positives are divided by real positives [57]. Recall is a measure of how many positive results the ML algorithms were able to produce [58]. The F1 score is calculated using the harmonic mean of precision and recall [57].


$$\begin{array}{c} \begin{array}{c} Accuracy=\frac{TP+TN}{TP+FP+FN+TN} \end{array} \end{array}$$ (10)

$$\begin{array}{c} \begin{array}{c} Precision=\frac{TP}{TP+FP} \end{array} \end{array}$$ (11)

$$\begin{array}{c} \begin{array}{c} Recall=\frac{TP}{TP+FN} \end{array} \end{array}$$ (12)

$$\begin{array}{c} \begin{array}{c} F\text{1}-Score=\frac{\text{2}*Precision*Recall}{Precision+Recall} \end{array} \end{array}$$ (13)

Kappa statistics are utilized to measure both observed and estimated accuracy [59,60]. In essence, Matthew’s correlation coefficient (MCC) is a correlation coefficient value between −1 and + 1 [58].


$$\begin{array}{c} \begin{array}{c} Kappa\;Statistics=\;\frac{observed\;accuracy-\;expected\;accuracy}{\text{1}-expected\;accuracy} \end{array} \end{array}$$ (14)

$$\begin{array}{c} \begin{array}{c} MCC=\;\frac{TP*TN-FP*FN}{\sqrt{\left(TP+FP)(TP+FN)(TN+FP)(TN+FN\right)}} \end{array} \end{array}$$ (15)

### 2.5. Features selection

Selecting the best features is a crucial stage of any ML approach; the selection of features determines the technique of exploring and selecting a subset of input features that are most related to the prediction feature [61]. Mutual information (MI) is a feature selection method based on information theory that employs IG for selecting the best features. The selection of MI features is suitable when the features are categorical or ordinal; at the same time, it also performs well for numerical features and categorical outcomes [62].

## 3. Result & discussion

The analysis results of the feature extractors and seven applied ML approaches that have been employed in this research to build an effective PPI prediction model have been described in this section.

### 3.1. Analysis of protein sequence

**Fig 2:**
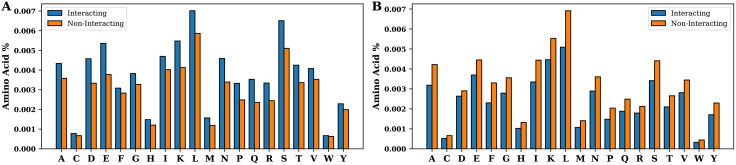
Amino acid percentages for protein sequences in the datasets Subplot ‘A’ for the amino acid percentages of the *S cerevisiae* dataset. And subplot ‘B’ for the amino acid percentages of the *H pylori* dataset.

Fig 2 illustrates the amino acid composition for both the *S cerevisiae* and *H pylori* datasets, highlighting notable trends in amino acid usage among interacting and non-interacting protein sequences. For both datasets, leucine (L) has the highest frequency among all amino acids, while tryptophan (W) is present in the lowest proportion. However, differences emerge when comparing the amino acid percentages between interacting and non-interacting sequences within each dataset: In the S. cerevisiae dataset, interacting sequences display a higher overall percentage of amino acids compared to non-interacting sequences, suggesting that amino acids are more abundant or frequent in protein sequences involved in interactions. Conversely, the *H pylori* dataset reveals a different pattern: non-interacting sequences have higher amino acid percentages than interacting ones. This implies that in *H pylori*, proteins not involved in interactions may exhibit a higher general amino acid composition compared to interacting proteins. These differences in amino acid composition between interacting and non-interacting sequences could reflect unique organism-specific features or protein interaction mechanisms within each dataset.

### 3.2. Performance evaluation of applied ML approaches

This study follows a two-stage analysis of the applied ML models. First, models were built using raw feature extraction methods, and the results for each method with the ML algorithms used are presented in Tables 1–5. Then, feature selection techniques were applied to the raw features, generating different subsets. The ML models were then built using these feature subsets, with the results shown in the Supplementary File (S1-S6 Tables) in S1 File. Upon comparing the performance of the raw feature extraction methods with that of the feature subsets, it was found that the raw feature encoding techniques consistently delivered better results. Consequently, the raw feature encoding methods were chosen for the final model development and performance evaluation.

**Table 1:** Result of the classifiers applied with the PAAC feature extractor on the *S cerevisiae* and *H pylori* data sets.

Dataset	ML Algorithm	Accuracy	MCC	Kappa	Precision	Recall	F1-Score
*S cerevisiae*	RF	0.9652	0.9308	0.9304	0.9652	0.9652	0.9652
	XGB	0.9532	0.9064	0.9063	0.9532	0.9532	0.9532
	CAT	0.9697	0.9395	0.9394	0.9697	0.9697	0.9697
	LGBM	0.9619	0.9240	0.9238	0.9619	0.9619	0.9619
	DT	0.8867	0.7733	0.7733	0.8867	0.8867	0.8867
	ETC	0.9043	0.8171	0.8108	0.9054	0.9054	0.9054
	SPPIPred	0.9736	0.9473	0.9473	0.9736	0.9736	0.9736
*H pylori*	RF	0.9420	0.8851	0.8841	0.9420	0.9420	0.9420
	XGB	0.9489	0.8979	0.8978	0.9489	0.9489	0.9489
	CAT	0.9516	0.9034	0.9033	0.9516	0.9516	0.9516
	LGBM	0.9509	0.9022	0.9019	0.9510	0.9510	0.9510
	DT	0.9287	0.8582	0.8573	0.9287	0.9287	0.9287
	ETC	0.9081	0.8233	0.8162	0.9081	0.9081	0.9081
	SPPIPred	0.9537	0.9075	0.9074	0.9537	0.9537	0.9537

**Table 2:** Result of the applied classifiers with the CTDC feature extractor in the *S cerevisiae* and *H pylori* datasets.

Dataset	ML Algorithm	Accuracy	MCC	Kappa	Precision	Recall	F1-Score
*S cerevisiae*	RF	0.9635	0.9271	0.9271	0.9635	0.9635	0.9635
	XGB	0.9502	0.9006	0.9004	0.9502	0.9502	0.9502
	CAT	0.9695	0.9391	0.9390	0.9695	0.9695	0.9695
	LGBM	0.9655	0.9311	0.9310	0.9655	0.9655	0.9655
	DT	0.9132	0.8267	0.8264	0.9132	0.9132	0.9132
	ETC	0.9176	0.8353	0.8351	0.9176	0.9176	0.9175
	SPPIPred	0.9725	0.9451	0.9451	0.9725	0.9725	0.9725
*H pylori*	RF	0.9503	0.9010	0.9005	0.9503	0.9503	0.9503
	XGB	0.9461	0.8924	0.8923	0.9461	0.9461	0.9461
	CAT	0.9516	0.9034	0.9033	0.9516	0.9516	0.9516
	LGBM	0.9492	0.8987	0.8985	0.9492	0.9492	0.9492
	DT	0.8813	0.7634	0.7627	0.8813	0.8813	0.8813
	ETC	0.9263	0.8542	0.8525	0.9263	0.9263	0.9263
	SPPIPred	0.9520	0.9040	0.9040	0.9520	0.9520	0.9520

**Table 3:** Result of the classifiers applied with the DPC feature extractor on the *S cerevisiae* and *H pylori* data sets.

Dataset	ML Algorithm	Accuracy	MCC	Kappa	Precision	Recall	F1-Score
*S cerevisiae*	RF	0.9630	0.9260	0.9260	0.9630	0.9630	0.9630
	XGB	0.9621	0.9243	0.9242	0.9621	0.9621	0.9621
	CAT	0.9663	0.9327	0.9326	0.9663	0.9663	0.9663
	LGBM	0.9633	0.9267	0.9265	0.9633	0.9632	0.9632
	DT	0.9296	0.8593	0.8593	0.9296	0.9296	0.9296
	ETC	0.9662	0.9324	0.9324	0.9662	0.9662	0.9662
	SPPIPred	0.9667	0.9333	0.9333	0.9667	0.9667	0.9667
*H pylori*	RF	0.9379	0.8761	0.8758	0.9379	0.9379	0.9379
	XGB	0.9520	0.9041	0.9040	0.9520	0.9520	0.9520
	CAT	0.9527	0.9054	0.9053	0.9527	0.9527	0.9527
	LGBM	0.9547	0.9096	0.9095	0.9547	0.9547	0.9547
	DT	0.9485	0.8971	0.8971	0.9485	0.9485	0.9485
	ETC	0.9184	0.8391	0.8368	0.9184	0.9184	0.9184
	SPPIPred	0.9523	0.9047	0.9047	0.9523	0.9523	0.9523

**Table 4:** Result of the classifiers applied with the Word2Vec feature extractor on the *S cerevisiae* and *H pylori* datasets.

Dataset	ML Algorithm	Accuracy	MCC	Kappa	Precision	Recall	F1-Score
*S cerevisiae*	RF	0.9500	0.9005	0.8999	0.9500	0.9500	0.9500
	XGB	0.9541	0.9084	0.9081	0.9541	0.9541	0.9541
	CAT	0.9413	0.8825	0.8824	0.9413	0.9413	0.9413
	LGBM	0.9534	0.9070	0.9067	0.9534	0.9534	0.9534
	DT	0.8949	0.7935	0.7889	0.8949	0.8949	0.8949
	ETC	0.9568	0.9137	0.9135	0.9568	0.9568	0.9568
	SPPIPred	0.9565	0.9132	0.9128	0.9565	0.9565	0.9565
*H pylori*	RF	0.9633	0.9274	0.9265	0.9633	0.9633	0.9633
	XGB	0.9718	0.9437	0.9437	0.9718	0.9718	0.9718
	CAT	0.9677	0.9356	0.9355	0.9677	0.9677	0.9677
	LGBM	0.9725	0.9451	0.9451	0.9725	0.9725	0.9725
	DT	0.9574	0.9154	0.9149	0.9574	0.9574	0.9574
	ETC	0.9729	0.9458	0.9458	0.9729	0.9729	0.9729
	SPPIPred	0.9681	0.9363	0.9362	0.9681	0.9681	0.9681

**Table 5:** Result of the classifiers applied with the FastText feature extractor in the *S cerevisiae* and *H pylori* datasets.

Dataset	ML Algorithm	Accuracy	MCC	Kappa	Precision	Recall	F1 score
*S cerevisiae*	RF	0.9990	0.9980	0.9980	0.9990	0.9990	0.9990
	XGB	0.9989	0.9978	0.9978	0.9989	0.9989	0.9989
	CAT	0.9986	0.9971	0.9971	0.9986	0.9986	0.9986
	LGBM	0.9989	0.9978	0.9978	0.9989	0.9989	0.9989
	DT	0.9802	0.9605	0.9605	0.9802	0.9802	0.9802
	ETC	0.9990	0.9980	0.9980	0.9990	0.9990	0.9990
	SPPIPred	0.9991	0.9982	0.9982	0.9991	0.9991	0.9991
*H pylori*	RF	0.9962	0.9925	0.9924	0.9962	0.9962	0.9962
	XGB	0.9986	0.9972	0.9972	0.9986	0.9986	0.9986
	CAT	0.9969	0.9938	0.9938	0.9969	0.9969	0.9969
	LGBM	0.9972	0.9945	0.9945	0.9972	0.9972	0.9972
	DT	0.9900	0.9801	0.9801	0.9900	0.9900	0.9900
	ETC	0.9797	0.9603	0.9595	0.9797	0.9797	0.9797
	SPPIPred	0.9989	0.9979	0.9979	0.9989	0.9989	0.9989

The dimensions of the extracted features vary across techniques: PAAC has a base dimension of 22, CTDC has 39, and DPC has 400. For Word2Vec and FastText, the feature dimensions are 512 in this study.

The results of all ML approaches with PAAC feature extractors on the two datasets obtained are presented in Table 1. According to Table 1, we can see that SPPIPred got the highest accuracy score for both datasets. The highest accuracy score for the *S cerevisiae* dataset is 0.9736, and the highest accuracy for the *H pylori* dataset is 0.9537. Although DT gives the lowest accuracy of 0.8867 among all classifiers on the S cerevisiae dataset, on the other hand, for the H pylori dataset, the lowest accuracy has been shown by ETC; the score is 0.9081. The maximum precision, recall, and f1 score result is the same as that obtained by SPPIPred, which is 0.9736. SPPIPred also received the highest MCC and Kappa scores, which are also the same; the value is 0.9473. These findings are obtained using the S cerevisiae data set. At the same time, in the H pylori data set, the maximum precision, recall, and f1 score are the same and have a value of 0.9537, which is obtained by SPPIPred. The highest MCC and Kappa scores are 0.9075 and 0.9074 for SPPIPred, respectively.

In Table 2, we present the results of the CTDC feature extractor with the seven applied ML classifiers on both datasets. Here, maximum accuracy, MCC, kappa, precision, recall, and F1-score have been achieved using the SPPIPred method of the *S cerevisiae* data set. The accuracy score is 0.9725. The MCC and Kappa scores are 0.9451. The precision, recall, and F1-score are all 0.9725. On the *H pylori* dataset, SPPIPred also obtained the highest results when applying performance metrics. MCC and Kappa both have scores of 0.9040. And the precision, recall, and f1 score are all the same, 0.9520. The accuracy result is 0.9520.

Table 3 shows the results of the DPC feature extractor with the different ML approaches in both the *S cerevisiae* and *H pylori* datasets. According to Table 3, the classifier with the highest accuracy on the *S cerevisiae* dataset is SPPIPred, which has an accuracy of 0.9667. Maximum precision, recall, and F1 score are 0.9667, which is the same for all three metrics. The highest Kappa and MCC scores are the same, which is 0.9333. On the other hand, for the *H. pylori dataset, the LGBM classifiers achieve the highest* accuracy. The LGBM precision score is 0.9547. LGBM also shows the highest result of all other metrics. MCC is 0.9096, kappa is 0.9095, precision is 0.9547 and recall and F1 score show the same precision result.

The results of the different classifiers that have been used in this study of the Word2Vec feature extractor for the two datasets are illustrated in Table 4. As shown in Table 4, the *S. cerevisiae* dataset shows the highest results of all performance metrics, by ETC. Precision, recall, and F1 score are all equal, yielding a value of 0.9568. The accuracy is 0.9568. MCC and Kappa values are 0.9137 and 0.9135, respectively. At the same time, on the *H pylori* data set, the ETC classifiers show the maximum results of all metrics, as well as an accuracy of 0.9729. Now, MCC and Kappa have the same value of 0.9458. In addition, precision, recall, and F1 score are also the same, which is 0.9729. Word2Vec is only the feature extractor for which the *H. pylori* dataset yields higher performance than the *S. cerevisiae* dataset.

The results of the FastText feature extractor with all applied ML classifiers are in Table 5. SPPIPred on both datasets produces the best results for all metrics that are used, as shown in Table 5. For the *S cerevisiae* dataset, the maximum accuracy is 0.9991. MCC and Kappa are both 0.9982. Precision, recall, and F1-score are all 0.9991. For the *H pylori* dataset, the highest accuracy is 0.9989. The MCC and kappa values are the same, that is, 0.9979. 0.9989 is the value of precision, recall, and F1-score. FastText is the feature extractor that has shown the highest results among all the feature extractors applied in this research.

**Fig 3:**
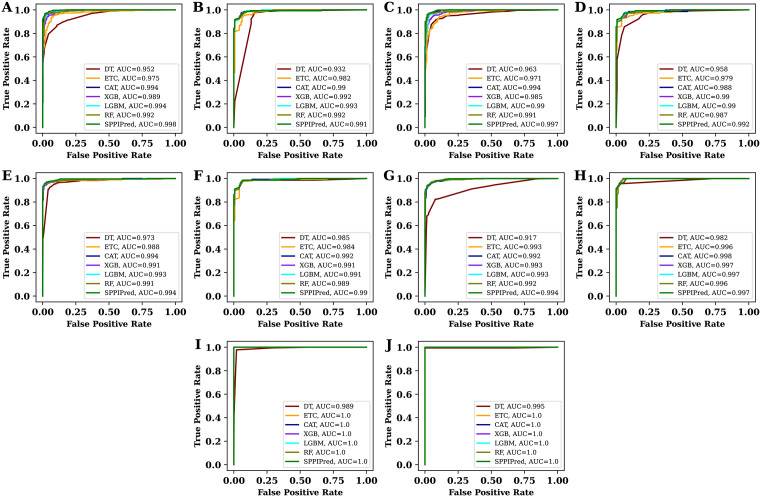
ROC curve of the applied classifiers based on the feature extraction method in the *S cerevisiae* and *H pylori* datasets. ROC curve (A-B) for the PAAC feature extractors of *S cerevisiae* (A) and *H pylori* (B). ROC curve (C-D) for the CTDC feature extractors of *S cerevisiae* (C) and *H pylori* (D). ROC curve (E-F) for the DPC feature extractors of *S cerevisiae* (E) and *H pylori* (F). ROC curve (G-H) for Word2Vec feature extractors of *S cerevisiae* (G) and *H pylori* (H). ROC curve (I-J) for the FastText feature extractors of *S cerevisiae* (I) and *H pylori* (J).

Fig 3 presents the ROC curves for different machine learning techniques applied to the *S cerevisiae* and *H pylori* datasets, using various feature extraction methods. For the *S cerevisiae* dataset, subplots A, C, E, G, and I show the performance of models trained with the feature extractors PAAC, CTDC, DPC, Word2Vec, and FastText, respectively. Among these, subplot I (FastText) shows the highest AUC score of 1.00 for the ETC, CAT, XGB, LGBM, RF, and SPPIPred models. The DT classifier, however, has a slightly lower AUC score of 0.987. Similarly, for the *H pylori* dataset, subplots B, D, F, H, and J illustrate the ROC curves for models using the PAAC, CTDC, DPC, Word2Vec, and FastText feature extractors, respectively. Here, subplot J (FastText) again shows the highest AUC scores, with all classifiers achieving an AUC of 1.000, except for the DT classifier, which reaches an AUC of 0.995. These results indicate that FastText is the most effective feature extractor in terms of AUC performance for both datasets, producing near-perfect classifier performance across most models.

### 3.3. Overall performance comparison of ML approaches and feature extractor with the data sets

**Fig 4:**
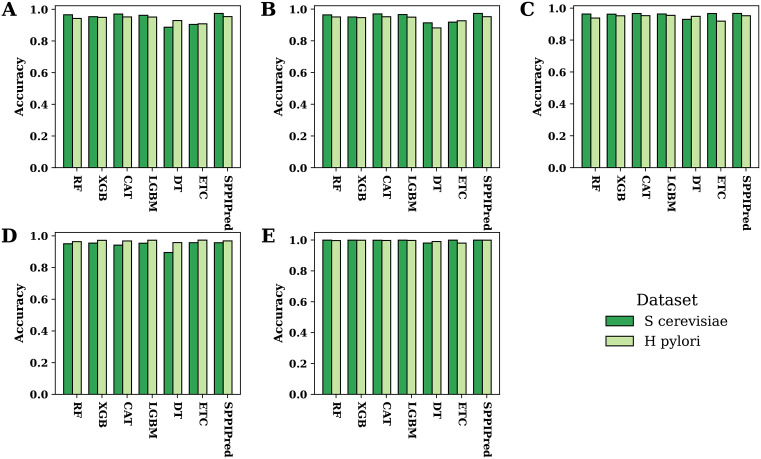
Performance comparison of the two data sets utilizing all the feature extractors applied. (A) The performance comparison of the PAAC features extractor. (B) Performance comparison of the CTDC feature extractor. (C) The performance comparison of the DPC features extractor. (D) The performance comparison of the Word2Vec features extractor. (E) The performance comparison of the FastText features extractor.

Fig 4 provides a comparative analysis of the model performance in the *S cerevisiae* and *H pylori* datasets, evaluating all machine learning classifiers and feature extractors. In general, the *S cerevisiae* dataset achieves superior performance in most feature extractors, except Word2Vec. For models trained with the PAAC feature extractor, the *S cerevisiae* dataset shows higher performance across all machine learning techniques, although DT and ETC perform slightly better on the *H pylori* dataset. Similarly, with the CTDC feature extractor, *S cerevisiae* demonstrates higher performance for most classifiers, except ETC, where the H. pylori dataset has an advantage. When using DPC and FastText feature extractors, the DT model performs better on the *H pylori* dataset, while other models still favor *S cerevisiae*. Interestingly, with the Word2Vec feature extractor, the *H pylori* data set outperforms *S cerevisiae* in all machine learning methods, highlighting the suitability of this feature extractor for the *H pylori* data set.

**Fig 5:**
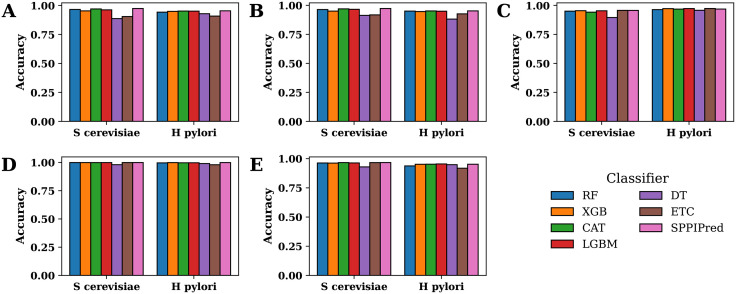
Comparison of performance among ML approaches based on two datasets and employing all feature extractors. (A) The results of the PAAC feature extractor. (B) Comparison of the CTDC feature extractor results. (C) The result of the Word2Vec feature extractor. (D) The comparison of the results of the FastText features extractor. (E) The result of the DPC feature extractor.

In Fig 5, subplots A (PAAC) and B (CTDC) show the highest accuracy achieved by the SPPIPred classifier on both datasets. ETC and SPPIPred show almost the same accuracy in the *S cerevisiae* dataset, but in the *H pylori* dataset, LGBM and ETC show nearly the same accuracy in Subplot-C (Word2Vec). Subplot-D (FastText) shows that, except for DT, the precision of all classifiers is nearly equal in the *S cerevisiae* dataset, for the *H pylori* data set, SPPIPred is maximum. In Subplot-E (DPC), ETC and SPPIPred performance is almost equally shown by the Fig, considering the *S cerevisiae* dataset. But for the *H pylori* dataset, the best results are shown by LGBM.

**Fig 6:**
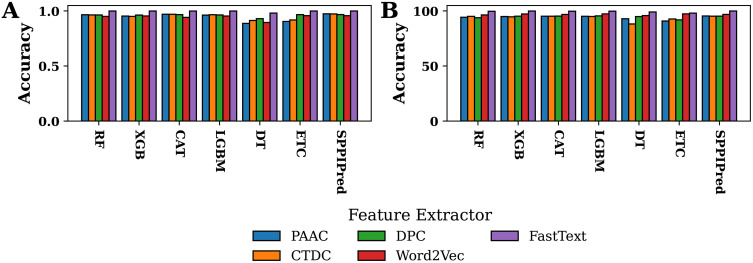
The performance comparison of all the feature extractors considers the ML approaches of two datasets. Subplot (A) is for the *S cerevisiae* dataset, and subplot (B) indicates the *H pylori* dataset.

Fig 6 illustrates a performance comparison among five feature extractors, evaluated separately in the *S cerevisiae* and *H pylori* datasets. In subplot A, representing *S cerevisiae*, the FastText feature extractor consistently outperforms the others across all machine learning models, demonstrating a marked improvement in model performance. Similarly, in subplot B, which represents *H pylori*, FastText again achieves the highest performance compared to the other feature extractors, showing its effectiveness for this data set as well. These results suggest that FastText is a highly effective feature extraction method for both data sets, improving model accuracy across various machine learning approaches.

### 3.4. Discussion

In recent years, numerous studies have focused on predicting protein-protein interactions (PPI) using machine learning (ML) techniques. Although significant progress has been made, there remains considerable potential for improvement in the accuracy and efficiency of PPI prediction models. Recognizing this need, the present research introduces a novel ML-based PPI prediction model. Machine learning has become a crucial tool in fields such as proteomics, bioinformatics, and systems biology due to its ability to overcome the limitations of traditional laboratory experiments, particularly in terms of reducing time, cost, and associated risks [63–65]. To develop the proposed PPI prediction model, this study utilized two benchmark protein sequence data sets: *S cerevisiae* and *H pylori*. After collecting the datasets, five distinct feature extraction techniques were applied to encode the protein sequences into feature vectors. These encoded features were then used to train and evaluate seven different ML classifiers. Among the feature extraction methods and classifiers tested, the FastText feature extractor combined with the proposed SPPIPred model demonstrated the best performance in both datasets. This suggests that the SPPIPred approach, using FastText for feature encoding, provides a more accurate and reliable solution for PPI prediction compared to other methods.

According to Table 6, SPPIPred clearly outperforms existing PPI prediction models on key metrics. Its accuracy is 0.0361 to 0.0548 higher, while the MCC is 0.0799 to 0.1085 higher, indicating better reliability. Precision also improves from 0.0179 to 0.0358, highlighting SPPIPred’s ability to more accurately identify positive interactions and reduce false positives. These results confirm SPPIPred’s superior performance in PPI prediction.

**Table 6:** For the *S cerevisiae* dataset, SPPIPred is compared with other existing PPI prediction models.

Model	Accuracy	MCC	Kappa	Precision
DeepPPI [66]	0.9443	0.8897	–	0.9665
LightGBM-PPI [67]	0.9507	0.9030	–	0.9782
StackPPI [4]	0.9464	0.8934	–	0.9633
GcForest-PPI [3]	0.9544	0.9102	–	0.9805
MARPPI [68]	0.9603	0.9183	–	0.9812
SPPIPred	0.9991	0.9982	0.9982	0.9991

According to Table 7, the SPPIPred model achieves substantial improvements over existing PPI prediction models, with improved accuracy from 0.0809 to 0.1366. Its MCC is 0.1605 to 0.2716 higher, indicating enhanced reliability and robustness. Additionally, the precision of SPPIPred surpasses that of other models by 0.092 to 0.1557, demonstrating better identification of positive interactions and fewer false positives. These results highlight SPPIPred’s superior overall performance compared to previous approaches.

**Table 7:** For the *H pylori* dataset, SPPIPred is compared with other exiting PPI prediction models.

Model	Accuracy	MCC	Kappa	Precision
DeepPPI [66]	0.8623	0.7263	–	0.8432
PCA-EELM [69]	0.8750	0.7813	–	0.8615
GcForest-PPI [3]	0.8926	0.7857	–	0.8895
StackPPI [4]	0.8927	0.7859	–	0.9037
MARPPI [68]	0.9180	0.8374	–	0.9069
SPPIPred	0.9989	0.9979	0.9979	0.9989

While the proposed PPI prediction model, SPPIPred, shows excellent performance, there are some limitations associated with the data sets used in this study. One limitation is the potential for bias in the data set, as the collected reference data sets may not fully represent the diversity of protein-protein interactions between different organisms. Additionally, the datasets might contain noise or incomplete annotations, which could affect the model’s ability to generalize effectively to unseen data. These factors could limit the broader applicability of the model and its performance on real-world PPI data. Additionally, although ML algorithms are effective, they may not fully exploit the complex relationships between proteins, which could affect the model’s generalization to diverse datasets. These limitations suggest that further exploration of more advanced methodologies could improve the robustness and accuracy of PPI predictions.

## 4. Conclusions

In this study, the prediction of PPI was addressed by proposing the SPPIPred model, which utilized five feature extraction methods (PAAC, CTDC, DPC, Word2Vec, and FastText) and seven machine learning algorithms (DT, ETC, CAT, XGB, LGBM, RF, and the stacking model SPPIPred).). FastText emerged as the top performance feature extractor, while the SPPIPred model achieved the best results in multiple evaluation metrics, including accuracy (0.9989 for *H pylori* and 0.9991 for *S cerevisiae*), as well as MCC and Kappa scores (0.9982 for *S cerevisiae* and 0.9979 for *H pylori*).). The findings of this research highlight the robustness and precision of the model, making it an invaluable tool in bioinformatics research, specifically for drug discovery, protein interaction network reconstruction, and signal transduction network construction. However, there are some limitations to this study that should be addressed in future work. Future studies could explore the incorporation of larger and more diverse datasets to improve the generalization of the model. In addition, incorporating deep learning approaches alongside more advanced feature extraction techniques could potentially enhance the accuracy and efficiency of the model’s prediction. By addressing these areas, future iterations of the SPPIPred model could offer even more precise and scalable solutions for protein-protein interaction prediction, benefiting both bioinformatics research and practical applications in bioengineering and drug development.

## Supporting information

S1 File Supporting tables. (DOCX)
