# Supplementary material for: SPPIPred: Stacking-based ensemble learning model for identification of protein-protein interaction
Source: PLoS One. 2026 Jul 17;21(7):e0353199. doi: 10.1371/journal.pone.0353199 (PMC13379025; doi:10.1371/journal.pone.0353199)
Supplement: S1 File — (DOCX) [file pone.0353199.s001.docx]

Table S1: Result of applied classifiers for Best selected features subset on CTDC (20&20).

| Dataset | ML Algorithm | Accuracy | MCC | Kappa | Precision | Recall | F1-Score |
| --- | --- | --- | --- | --- | --- | --- | --- |
| *S cerevisiae* | RF | 0.9599 | 0.9201 | 0.9199 | 0.9599 | 0.9599 | 0.9599 |
|  | XGB | 0.9422 | 0.8844 | 0.8843 | 0.9422 | 0.9422 | 0.9422 |
|  | CAT | 0.9695 | 0.9391 | 0.9390 | 0.9695 | 0.9695 | 0.9695 |
|  | LGBM | 0.9603 | 0.9207 | 0.9206 | 0.9603 | 0.9603 | 0.9603 |
|  | DT | 0.9078 | 0.8159 | 0.8157 | 0.9078 | 0.9078 | 0.9078 |
|  | ETC | 0.8888 | 0.7778 | 0.7776 | 0.8888 | 0.8888 | 0.8888 |
|  | SPPIPred | 0.9734 | 0.9468 | 0.9467 | 0.9734 | 0.9734 | 0.9734 |
| *H pylori* | RF | 0.9523 | 0.9049 | 0.9047 | 0.9523 | 0.9523 | 0.9523 |
|  | XGB | 0.9461 | 0.8923 | 0.8923 | 0.9461 | 0.9461 | 0.9461 |
|  | CAT | 0.9516 | 0.9034 | 0.9033 | 0.9516 | 0.9516 | 0.9516 |
|  | LGBM | 0.9503 | 0.9005 | 0.9005 | 0.9503 | 0.9503 | 0.9503 |
|  | DT | 0.9002 | 0.8004 | 0.8004 | 0.9002 | 0.9002 | 0.9002 |
|  | ETC | 0.9029 | 0.8114 | 0.8059 | 0.9029 | 0.9029 | 0.9029 |
|  | SPPIPred | 0.9499 | 0.8998 | 0.8999 | 0.9499 | 0.9499 | 0.9499 |

Table S2: Result of applied classifiers for Best selected features subset on DPC (50&20).

| Dataset | ML Algorithm | Accuracy | MCC | Kappa | Precision | Recall | F1-Score |
| --- | --- | --- | --- | --- | --- | --- | --- |
| *S cerevisiae* | RF | 0.9507 | 0.9013 | 0.9013 | 0.9507 | 0.9507 | 0.9507 |
|  | XGB | 0.9520 | 0.9041 | 0.9040 | 0.9520 | 0.9520 | 0.9520 |
|  | CAT | 0.9654 | 0.9309 | 0.9308 | 0.9654 | 0.9654 | 0.9654 |
|  | LGBM | 0.9541 | 0.9084 | 0.9083 | 0.9541 | 0.9541 | 0.9541 |
|  | DT | 0.9273 | 0.8567 | 0.8547 | 0.9273 | 0.9273 | 0.9273 |
|  | ETC | 0.9470 | 0.8940 | 0.8940 | 0.9470 | 0.9470 | 0.9470 |
|  | SPPIPred | 0.9674 | 0.9348 | 0.9347 | 0.9674 | 0.9674 | 0.9674 |
| *H pylori* | RF | 0.9523 | 0.9047 | 0.9047 | 0.9523 | 0.9523 | 0.9523 |
|  | XGB | 0.9472 | 0.8945 | 0.8944 | 0.9472 | 0.9472 | 0.9472 |
|  | CAT | 0.9520 | 0.9041 | 0.9039 | 0.9520 | 0.9520 | 0.9520 |
|  | LGBM | 0.9509 | 0.9019 | 0.9019 | 0.9509 | 0.9509 | 0.9509 |
|  | DT | 0.9376 | 0.8756 | 0.8752 | 0.9376 | 0.9376 | 0.9376 |
|  | ETC | 0.9407 | 0.8816 | 0.8813 | 0.9407 | 0.9407 | 0.9407 |
|  | SPPIPred | 0.9534 | 0.9067 | 0.9067 | 0.9534 | 0.9534 | 0.9534 |

Table S3: Result of applied classifiers for Best selected features subset on Word2Vec (100&50).

| Dataset | ML Algorithm | Accuracy | MCC | Kappa | Precision | Recall | F1-Score |
| --- | --- | --- | --- | --- | --- | --- | --- |
| *S cerevisiae* | RF | 0.9295 | 0.8590 | 0.8590 | 0.9295 | 0.9295 | 0.9295 |
|  | XGB | 0.9456 | 0.8913 | 0.8911 | 0.9456 | 0.9456 | 0.9456 |
|  | CAT | 0.9266 | 0.8530 | 0.8531 | 0.9266 | 0.9266 | 0.9266 |
|  | LGBM | 0.9425 | 0.8849 | 0.8849 | 0.9425 | 0.9425 | 0.9425 |
|  | DT | 0.8996 | 0.7997 | 0.7988 | 0.8996 | 0.8996 | 0.8996 |
|  | ETC | 0.9561 | 0.9122 | 0.9121 | 0.9561 | 0.9561 | 0.9561 |
|  | SPPIPred | 0.9569 | 0.9142 | 0.9137 | 0.9569 | 0.9569 | 0.9569 |
| *H pylori* | RF | 0.9636 | 0.9277 | 0.9273 | 0.9636 | 0.9636 | 0.9636 |
|  | XGB | 0.9715 | 0.9430 | 0.9430 | 0.9715 | 0.9715 | 0.9715 |
|  | CAT | 0.9694 | 0.9389 | 0.9389 | 0.9694 | 0.9694 | 0.9694 |
|  | LGBM | 0.9722 | 0.9444 | 0.9444 | 0.9722 | 0.9722 | 0.9722 |
|  | DT | 0.9612 | 0.9226 | 0.9224 | 0.9612 | 0.9612 | 0.9612 |
|  | ETC | 0.9739 | 0.9479 | 0.9478 | 0.9739 | 0.9739 | 0.9739 |
|  | SPPIPred | 0.9677 | 0.9356 | 0.9354 | 0.9677 | 0.9677 | 0.9677 |

Table S4: Result of applied classifiers for Best selected features subset on FastText (50&100).

| Dataset | ML Algorithm | Accuracy | MCC | Kappa | Precision | Recall | F1-Score |
| --- | --- | --- | --- | --- | --- | --- | --- |
| *S cerevisiae* | RF | 0.9956 | 0.9912 | 0.9912 | 0.9956 | 0.9956 | 0.9956 |
|  | XGB | 0.9971 | 0.9943 | 0.9947 | 0.9971 | 0.9971 | 0.9971 |
|  | CAT | 0.9969 | 0.9939 | 0.9939 | 0.9969 | 0.9969 | 0.9969 |
|  | LGBM | 0.9981 | 0.9962 | 0.9962 | 0.9981 | 0.9981 | 0.9981 |
|  | DT | 0.9725 | 0.9453 | 0.9450 | 0.9725 | 0.9725 | 0.9725 |
|  | ETC | 0.9809 | 0.9619 | 0.9619 | 0.9809 | 0.9809 | 0.9809 |
|  | SPPIPred | 0.9985 | 0.9969 | 0.9969 | 0.9985 | 0.9985 | 0.9985 |
| *H pylori* | RF | 0.9914 | 0.9829 | 0.9829 | 0.9914 | 0.9914 | 0.9914 |
|  | XGB | 0.9959 | 0.9918 | 0.9918 | 0.9959 | 0.9959 | 0.9959 |
|  | CAT | 0.9979 | 0.9959 | 0.9959 | 0.9979 | 0.9979 | 0.9979 |
|  | LGBM | 0.9962 | 0.9924 | 0.9924 | 0.9962 | 0.9962 | 0.9962 |
|  | DT | 0.9708 | 0.9421 | 0.9417 | 0.9708 | 0.9708 | 0.9708 |
|  | ETC | 0.9698 | 0.9413 | 0.9396 | 0.9698 | 0.9698 | 0.9698 |
|  | SPPIPred | 0.9972 | 0.9945 | 0.9945 | 0.9972 | 0.9972 | 0.9972 |

Table S5: Result of applied classifiers on Combined features.

| Dataset | ML Algorithm | Accuracy | MCC | Kappa | Precision | Recall | F1-Score |
| --- | --- | --- | --- | --- | --- | --- | --- |
| *S cerevisiae* | RF | 0.9973 | 0.9946 | 0.9946 | 0.9973 | 0.9973 | 0.9973 |
|  | XGB | 0.9969 | 0.9937 | 0.9937 | 0.9969 | 0.9969 | 0.9969 |
|  | CAT | 0.9978 | 0.9955 | 0.9955 | 0.9978 | 0.9978 | 0.9978 |
|  | LGBM | 0.9973 | 0.9946 | 0.9946 | 0.9973 | 0.9973 | 0.9973 |
|  | DT | 0.9756 | 0.9512 | 0.9511 | 0.9756 | 0.9756 | 0.9756 |
|  | ETC | 0.9966 | 0.9932 | 0.9932 | 0.9966 | 0.9966 | 0.9966 |
|  | SPPIPred | 0.9978 | 0.9955 | 0.9955 | 0.9978 | 0.9978 | 0.9978 |
| *H pylori* | RF | 0.9942 | 0.9883 | 0.9883 | 0.9942 | 0.9942 | 0.9942 |
|  | XGB | 0.9966 | 0.9931 | 0.9931 | 0.9966 | 0.9966 | 0.9965 |
|  | CAT | 0.9962 | 0.9924 | 0.9924 | 0.9962 | 0.9962 | 0.9962 |
|  | LGBM | 0.9959 | 0.9918 | 0.9918 | 0.9959 | 0.9959 | 0.9959 |
|  | DT | 0.9869 | 0.9739 | 0.9739 | 0.9869 | 0.9869 | 0.9869 |
|  | ETC | 0.9921 | 0.9843 | 0.9842 | 0.9921 | 0.9921 | 0.9921 |
|  | SPPIPred | 0.9972 | 0.9945 | 0.9945 | 0.9972 | 0.9972 | 0.9972 |

Table S6: Result of applied classifiers on Combined Best subset after features selection (100&20).

| Dataset | ML Algorithm | Accuracy | MCC | Kappa | Precision | Recall | F1-Score |
| --- | --- | --- | --- | --- | --- | --- | --- |
| *S cerevisiae* | RF | 0.9713 | 0.9428 | 0.9425 | 0.9713 | 0.9713 | 0.9713 |
|  | XGB | 0.9798 | 0.9596 | 0.9595 | 0.9798 | 0.9798 | 0.9798 |
|  | CAT | 0.9919 | 0.9839 | 0.9839 | 0.9919 | 0.9919 | 0.9919 |
|  | LGBM | 0.9905 | 0.9810 | 0.9810 | 0.9905 | 0.9905 | 0.9905 |
|  | DT | 0.9338 | 0.8685 | 0.8674 | 0.9338 | 0.9338 | 0.9338 |
|  | ETC | 0.9876 | 0.9751 | 0.9751 | 0.9876 | 0.9876 | 0.9876 |
|  | SPPIPred | 0.9956 | 0.9912 | 0.9912 | 0.9956 | 0.9956 | 0.9956 |
| *H pylori* | RF | 0.9887 | 0.9773 | 0.9773 | 0.9887 | 0.9887 | 0.9887 |
|  | XGB | 0.9945 | 0.9890 | 0.9890 | 0.9945 | 0.9945 | 0.9945 |
|  | CAT | 0.9948 | 0.9897 | 0.9897 | 0.9948 | 0.9948 | 0.9948 |
|  | LGBM | 0.9969 | 0.9938 | 0.9938 | 0.9969 | 0.9969 | 0.9969 |
|  | DT | 0.9753 | 0.9507 | 0.9506 | 0.9753 | 0.9753 | 0.9753 |
|  | ETC | 0.9918 | 0.9835 | 0.9835 | 0.9918 | 0.9918 | 0.9918 |
|  | SPPIPred | 0.9955 | 0.9910 | 0.9911 | 0.9955 | 0.9955 | 0.9955 |
